# The secreted APP ectodomain sAPPα, but not sAPPβ, protects neurons against Aβ oligomer-induced dendritic spine loss and increased tau phosphorylation

**DOI:** 10.1186/s13041-019-0447-2

**Published:** 2019-03-29

**Authors:** Christian Tackenberg, Roger M. Nitsch

**Affiliations:** 1 Institute for Regenerative Medicine, University of Zurich, Schlieren, Switzerland; 2 Neuroscience Center Zurich, University of Zurich, Zurich, Switzerland

**Keywords:** sAPPα, sAPPβ, Aβ, Tau, Dendritic spines, Alzheimer’s disease

## Abstract

**Aim:**

The amyloid precursor protein (APP) is endoproteolytically processed to generate either the neurotoxic beta-amyloid peptide (Aβ) or the secreted ectodomain APP alpha (sAPPα). While neurotrophic properties of sAPPα were suggested in several studies, it is still unclear if and how sAPPα counteracts pathogenic effects of Aβ. Direct comparisons with sAPPβ, produced in the Aβ-generating pathway, are missing in order to determine the role of sAPPα’s carbonyl-terminal end in its possible neuroprotective activity.

**Methods:**

Mouse neuronal primary cultures and hippocampal slices were treated with oligomeric Aβ_42_. The effects on tau phosphorylation and dendritic spine densities were assessed by western blot and confocal imaging, respectively. Co-administration of either sAPPα or sAPPβ was used to determine activity on Aβ-induced toxicity.

**Results/discussion:**

We found that oligomeric Aβ strongly increased AT8 and AT180 phosphorylation of tau and caused a loss of dendritic spines. SAPPα completely abolished Aβ effects whereas sAPPβ had no such rescue activity. Interestingly, sAPPα or sAPPβ alone neither affected tau phosphorylation nor dendritic spine numbers. Together, our data suggest that sAPPα specifically protects neurons against Aβ-dependent toxicity supporting the strategy of activating α-secretase-dependent endoproteolytic APP processing to increase sAPPα shedding from the neuronal plasma membrane as a therapeutic intervention for the protection of dendritic spines and phospho-tau-dependent toxicity in Alzheimer’s disease.

**Electronic supplementary material:**

The online version of this article (10.1186/s13041-019-0447-2) contains supplementary material, which is available to authorized users.

## Main text

The depositions of Aβ and hyperphosphorylated tau are the major hallmarks of Alzheimer’s disease (AD). Aβ is produced by the amyloidogenic processing of APP. In this pathway, APP is cleaved by β-secretase (BACE-1) generating soluble APPβ (sAPPβ) and the membrane-bound fragment β-CTF. Further processing of β-CTF by γ-secretase produces the APP intracellular domain (AICD) and Aβ forms of various lengths. In the non-amyloidogenic pathway, Aβ generation is precluded by α-secretase, i.e. the metalloproteases ADAM10 and ADAM17, which cleave within the Aβ sequence generating sAPPα and α-CTF. Subsequent γ-secretase cleavage releases AICD and the P3 fragment (reviewed in [1]).

Most AD-related studies focus on the detrimental effect of Aβ and therapeutic interventions aim in reducing Aβ levels. However, sAPP, especially sAPPα, is thought to have neurotrophic and neuroprotective properties [2] and its levels are strongly reduced in AD patients with one of two copies of APOE4, the main risk factor for AD. Interestingly, the levels of sAPPβ were not different in AD patients compared to controls [3]. In contrast to sAPPα, sAPPβ is not or much less potent in neurotrophic support [2]. In several studies on the beneficial or detrimental effects of sAPPα or sAPPβ, the direct comparison of both is lacking. Here we aim to explore the neuroprotective properties of sAPPα on Aβ-induced neuronal dysfunction in direct comparison to sAPPβ.

**Fig. 1:**
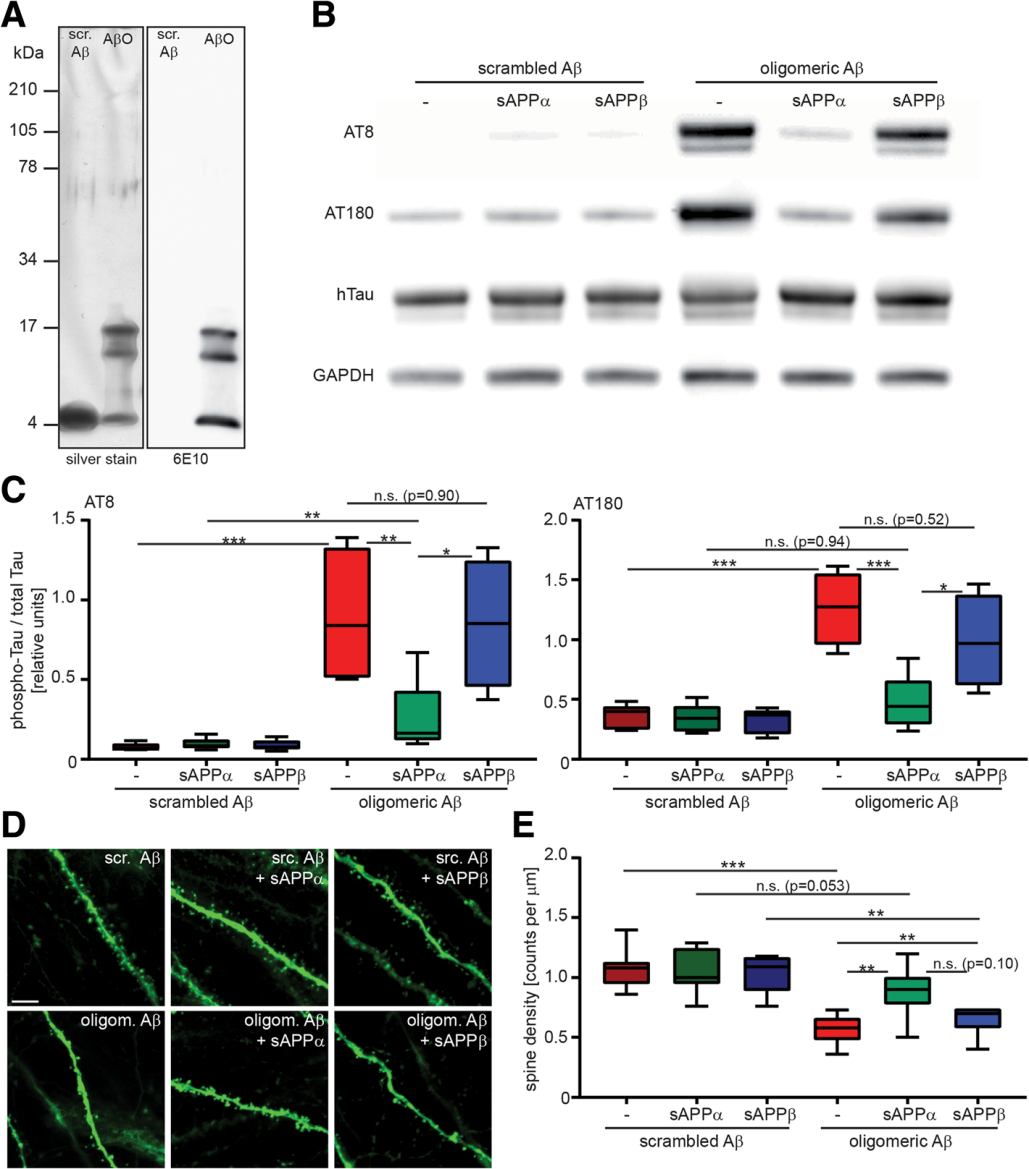
Treatment with sAPPα but not sAPPβ reduces Aβ-induced tau phosphorylation and dendritic spine loss. **a** Oligomeric Aβ preparations were characterized by SDS-PAGE followed by silver staining (left panel) or western blot using 6E10 antibody (right panel). Besides monomers, trimers and tetramers were the main Aβ species obtained by the used oligomerization protocol. **b** Primary neuronal cultures overexpressing human 441 tau were treated with 500 nM oligomeric Aβ or scrambled control and 400 ng/ml sAPPα or sAPPβ. AT8 and AT180 phosphorylation was detected in cell lysates. **c** Quantification of western blots. Aβ oligomers increased AT8 and AT180 phosphorylation of tau which was prevented by 400 ng/ml sAPPα but not sAPPβ. Statistical comparison between multiple groups was performed using one-way ANOVA with Tukey’s test for multiple comparisons *n* = 5 (*, *p* < 0,05; **, *p* > 0,01; ***, *p* < 0.001;). For important non-significant results, the exact *p*-values are displayed. **d** Representative images of CA1 apical dendritic segments of organotypic hippocampal slice cultures expressing eGFP. Slices were treated with 500 nM oligomeric Aβ or scrambled control and 400 ng/ml sAPPα or sAPPβ. scale bar = 5 μm **e** Analysis of dendritic spine density displayed as spine counts per μm dendrite. Spine density was strongly reduced by Aβ treatment. sAPPα but not sAPPβ diminished dendritic spine loss caused by Aβ. Statistical comparison between multiple groups was performed using one-way ANOVA with Tukey’s test for multiple comparisons *n* = 7–11, Hippocampal slices were prepared from at least 3 different mice per condition. (**, *p* > 0,01; ***, *p* < 0.001;). For important non-significant results, the exact *p*-values are displayed. Scr. Aβ: scrambled Aβ; AβO: oligomeric Aβ

Oligomeric Aβ induces hyperphosphorylation of tau, which can cause toxicity downstream of Aβ [4]. To determine a potential effect of sAPPα on Aβ-induced tau phosphorylation primary neuronal cultures were transduced with neurotropic Sindbis virus to express the 441 amino acid isoform of human tau selectively in neurons [5]. Furthermore, cultures were treated with 500 nM oligomeric Aβ_42_ or scrambled Aβ_42_ (Additional file 1). Preparations of Aβ_42_ oligomers mainly consist of mono-, tri- and tetramers (Fig. 1a), which have been shown before to be cause neuronal dysfunctions [6]. As expected, treatment with oligomeric Aβ strongly increased tau phosphorylation at the AD-relevant epitopes AT8 and AT180 (Fig. 1b and c). The AT8 antibody detects phosphorylation at Ser202/Thr205, AT180 binds specifically to phosphorylated Thr231. Both epitopes are target of the main tau kinase, glycogen synthase kinase 3 beta (GSK-3β) [7]. Co-treatment of cultures with 400 ng/ml recombinant sAPPα strongly reduced Aβ-induced tau phosphorylation. In contrast, treatment with sAPPβ had no effect on tau phosphorylation in the presence of Aβ (Fig. 1b and c).

In addition, we analyzed whether sAPPα and sAPPβ can modulate dendritic spine densities, as synapse loss strongly correlates with the degree of dementia in AD. To this end, we used organotypic slice cultures which were transduced with Sindbis virus to express enhanced GFP (EGFP). This method has been described before and allows morphological analysis of neuronal connectivity in slices [5, 6]. As previous studies showed that the expression of human tau in this system does not affect dendritic spine number or morphology, analysis of spines was performed in the absence of tau expression for this study [5, 8]. Images of dendritic segments in CA1 *stratum radiatum* were analyzed for dendritic spine density (Fig. 1d). CA1 s*tratum radiatum* apical dendrites were chosen as they allow reliable imaging and evaluation due to the presence of long and straight dendritic segments. Further, Aβ affects different hippocampal regions, such as CA1 and CA3, to a similar extent [5]. We and others have shown that Aβ reduces the density of postsynaptic spines and alters their morphology in slice cultures (reviewed in [9]). Accordingly, treatment of slices with oligomeric Aβ but not scrambled Aβ strongly reduced dendritic spine numbers (Fig. 1e). We then determined if sAPPα or sAPPβ may prevent spine loss. The presence of 400 ng/ml sAPPα completely abolished Aβ-induced spine loss while sAPPβ-treated slices still displayed a significant spine reduction (Fig. 1e). To the best of our knowledge, this is the first report of a protective mechanism of sAPPα for Aβ-induced dendritic spine loss.

The CSF levels of sAPP in human patients reported in the literature strongly vary among different studies ranging from approx. 0,55 ng/ml and 0,25 ng/ml to 1800 ng/ml and 1600 ng/ml for sAPPα and sAPPβ, respectively [3, 10]. Also, the ratios between sAPPα and sAPPβ vary. For our analyses we used equimolar levels of sAPPα and sAPPβ at concentrations within the range described in the literature. It is important to note that both, sAPPα and sAPPβ alone, neither affect tau phosphorylation nor dendritic spine numbers. Thus, the effect of sAPPα represents a specific protective mechanism against Aβ-induced neuronal dysfunctions rather than a general neurotrophic effect.

sAPPα reduced Aβ-induced tau phosphorylation by increasing the expression of the Aβ-binding protein transthyretin (TTR) [11]. However, the Aβ concentrations used in that study were very high (50 μM) and no comparison with sAPPβ was performed. Since we used sAPPβ as control we can clearly show that the protective property of sAPPα lies within the C-terminal part of the peptide. Another study suggested that sAPPα reduces tau phosphorylation by GSK-3β inhibition [12]. However, we did not observe a reduction in tau phosphorylation by treatment with sAPPα in the absence of Aβ oligomers. This implies that either sAPPα does not inhibit GSK-3β in our model or that this inhibition only becomes noticeable after an activation of GSK-3β by Aβ. Thus, it may be interesting to investigate the potential protective mechanism in our model in a future study in more detail.

Increasing sAPPα levels by activating α-secretase, specifically ADAM10, is of therapeutic potential for the treatment of neurodegenerative conditions including AD. Accordingly, mild overexpression of ADAM10 prevented amyloid plaque formation and hippocampal defects in transgenic AD mice [13]. However, ADAM10 is a widely distributed transmembrane protease and involved in shedding many different substrates. Clinical studies are required to determine whether therapeutic benefits of α-secretase activation would outweigh potential side effects (reviewed in [14]).

Another current strategy is the pharmacological reduction of Aβ production by inhibition of γ-secretase using γ-secretase inhibitors or modulators (GSIs, GSMs). Recently, it was shown that inhibition of γ-secretase activity activated a feedback loop leading to increased α-secretase processing and accelerated release of sAPPα [15]. Thus, GSIs may act via a dual protective mechanism, reduction of neurotoxic Aβ and elevation of neuroprotective sAPPα levels.

Taken together, restoration of sAPPα by increasing non-amyloidogenic processing can reduce Aβ pathology and therefore represents a valid therapeutic approach for AD (Additional file 1).

## Additional file

Additional file 1: Soluble APPα but not soluble APPβ protects against Aβ oligomer-induced dendritic spine loss and increased Tau phosphorylation. (PDF 143 kb)

## Abbreviations

AD: Alzheimer’s disease
ADDLs: Aβ-derived diffusible ligands
APP: Amyloid precursor protein
Aβ: Beta-amyloid peptide
AβO: Oligomeric Aβ
GSIs: γ-secretase inhibitors
GSK-3β: Glycogen synthase kinase 3 beta
sAPP: Soluble APP
Scr. Aβ: Scrambled Aβ
TTR: Transthyretin
