# Supplementary material for: The secreted APP ectodomain sAPPα, but not sAPPβ, protects neurons against Aβ oligomer-induced dendritic spine loss and increased tau phosphorylation
Source: Mol Brain. 2019 Mar 29;12:27. doi: 10.1186/s13041-019-0447-2 (PMC6440141; doi:10.1186/s13041-019-0447-2)
Supplement: Supplementary file 1 — Soluble APPα but not soluble APPβ protects against Aβ oligomer-induced dendritic spine loss and increased Tau phosphorylation. (PDF 143 kb) [file 13041_2019_447_MOESM1_ESM.pdf]

Additional file 1

Soluble APPα but not soluble APPβ protects against Aβ oligomer-induced dendritic spine loss and increased Tau phosphorylation

Christian Tackenberg[1,2,*] and Roger M. Nitsch[1]

[1]Institute for Regenerative Medicine, University of Zurich, Schlieren, Switzerland

[2]Neuroscience Center Zurich, University of Zurich, Zurich, Switzerland

* Corresponding author

Materials and Methods

Aβ-oligomer preparation

Synthetic Aβ42 peptide was obtained from American peptide. Preparation of Aβ42 oligomers (ADDLs) was carried out as previously described (1). In short, cold 1,1,1,3,3,3-hexafluro-2-propanol (HFIP) was added to Aβ42 peptide to a final concentration of 1 mM. HFIP was evaporated overnight and peptides dried for 10 min in a speedvac and stored at -80°C. For experiments, Aβ42 peptide was resuspended in DMSO at a concentration of 5 mM. Neurobasal medium without phenol red (Gibco) was added to achieve a peptide concentration of 100 µM. The solution was incubated for 24 h at 4°C. Higher aggregates e.g. fibrils were removed by centrifugation at 14.000 g for 10 minutes at 4°C and the supernatant was used for experimental procedures. Aβ42 oligomer preparations were routinely analyzed by silver staining and western blot for each experiment (Fig. 1A).

Primary neuronal cell culture

Primary neurons were prepared from E16 C57BL/6JOlaHsd mice (Harlan). Cells were plated in 6 well dishes at a density of 2.5 x 10^5 cells/well. Cultures were maintained in Neurobasal medium supplemented with B27, GlutaMAX and 500 µM sodium pyruvate at 37°C and 7% CO2. On DIV 18 neurons were transduced with Sindbis virus expressing the EGFP-coupled 441 amino acid isoform of

human Tau as previously described (2). Additionally, cells were treated with 500 nM oligomeric Aβ and 400 ng/ml sAPPα or sAPPβ respectively. The oligomerization protocol was also applied to 500 nM scrambled Aβ serving as control. 16 h after transduction and treatments cells were lysed in RIPA buffer (50 mM Tris-HCl, 150 mM NaCl, 2 mM EDTA, 1% NP-40, 0.5% deoxycholate, 0.1% SDS, pH 8.0) containing protease and phosphatase inhibitors, sonicated and centrifuged for 10 min 14000g at 4°C. Lysates were analyzed for phosphorylation levels of human Tau using western blot.

Organotypic hippocampal slice cultures

The hippocampus of one-week-old C57BL/6JOlaHsd mice (Harlan) was dissected and cut into 400 µm thick slices. These were cultured on Millicell culture plate inserts (Millipore) for 15 DIV in culture medium (Minimum essential medium Eagle with HEPES modification, 25% basal medium with Earle's modification, 25% heat-inactivated horse serum, 2 mM glutamine, 0.6% glucose, 50U/ml Penicillin-Streptomycin, pH 7.2). Medium was changed every second to third day. Cell culture reagents were purchased from Gibco, Life Technologies and SIGMA. All animal experiments were performed in accordance with the guidelines of the Swiss veterinary cantonal office. To assess the effect of Aβ oligomers, sAPPα and sAPPβ on dendritic spines, slices were treated with 500 nM oligomeric Aβ or scrambled Aβ control and 400 ng/ml recombinant sAPPα or sAPPβ (Meso Scale Diagnostics) from DIV 11-15.

Analysis of dendritic spine density

Hippocampal slice cultures were infected on DIV 12 with Sindbis virus expressing EGFP and fixed on DIV15 with 4% paraformaldehyde in PBS containing 4% sucrose for 2 h at 4°C. For analysis of dendritic spine density, virus solution was diluted to achieve 1–10 infected neurons per slice. This allowed imaging of single dendritic fragments. Analysis of dendritic spine density was performed using Leica SP2 CLSM equipped with 63x objective (NA: 1.2) and 488-nm Argon laser. Images of apical dendritic segments in CA1 *stratum radiatum* were acquired with image settings of 512 x 512 pixel and voxel size of 0.05813 x 0.05813 x 0.25 µm. Image stacks were processed to maximum projections, and dendritic spine density was determined as spine counts per µm dendrite using ImageJ.

Westernblot

Samples were resolved by 10–20% SDS-PAGE and transferred to nitrocellulose membranes. Immunoblotting was performed using primary antibodies anti-GFP (1:1000, Roche) to detect total EGFP-coupled human Tau, anti phospho-Tau AT8 (1:200, Thermo Fisher) and AT180 (1:500, Thermo Fisher), GAPDH (1:5000, Biodesign) and HRP-conjugated secondary antibody (1:2000, GE Healthcare). Immunoreactive bands were detected using the ECL Reagent (Thermo Fisher) according to the manufacturer's instructions and imaged with Fujifilm Las3000. It was verified by software tools that no pixels were saturated. Band intensities were quantified with ImageJ corrected by background.

Silver staining

To analyze oligomeric Aβ42 preparations, samples were run on a 10-20% Tricine polyacrylamide gel and the gel was subsequently left overnight in fixing solution (40% EtOH, 10% acetic acid). After washing in H_20, the gel was sensitized in 0.017% sodium thiosulfate for 2 min, washed and impregnated in 0.27% silver nitrate solution (including 0.37% formaldehyde) for 30 min. After rinsing in H_20 the gel was developed in 0.03 M sodium carbonate (to which 0.15% Formaldehyde and 0.02% sodium thiosulfate was added). The reaction was stopped with 3% glacial acid.

References

1. Klein WL. Abeta toxicity in Alzheimer's disease: globular oligomers (ADDLs) as new vaccine and drug targets. Neurochem Int. 2002;41:345-52.
2. Tackenberg C, Grinschgl S, Trutzel A, Santuccione AC, Frey MC, Konietzko U, et al. NMDA receptor subunit composition determines beta-amyloid-induced neurodegeneration and synaptic loss. Cell Death Dis. 2013;4:e608.
